# Overload-induced skeletal muscle hypertrophy is not impaired in STZ-diabetic rats

**DOI:** 10.14814/phy2.12457

**Published:** 2015-07-21

**Authors:** Marco Aurélio S Fortes, Carlos Hermano J Pinheiro, Lucas Guimarães-Ferreira, Kaio F Vitzel, Diogo A A Vasconcelos, Rui Curi

**Affiliations:** 1 Department of Physiology and Biophysics, Institute of Biomedical Sciences, University of São PauloSão Paulo, Brazil; 2 Exercise Metabolism Research Group, Center of Physical Education and Sports, Federal University of Espírito SantoVitória, Brazil

**Keywords:** Diabetes *mellitus*, electrical stimulation, hyperglycemia, muscle mass regulation, streptozotocin

## Abstract

The aim of this study was to evaluate the effect of overload-induced hypertrophy on extensor digitorum longus (EDL) and soleus muscles of streptozotocin-induced diabetic rats. The overload-induced hypertrophy and absolute tetanic and twitch forces increases in EDL and soleus muscles were not different between diabetic and control rats. Phospho-Akt and rpS6 contents were increased in EDL muscle after 7 days of overload and returned to the pre-overload values after 30 days. In the soleus muscle, the contents of total and phospho-Akt and total rpS6 were increased in both groups after 7 days. The contents of total Akt in controls and total rpS6 and phospho-Akt in the diabetic rats remained increased after 30 days. mRNA expression after 7 days of overload in the EDL muscle of control and diabetic animals showed an increase in MGF and follistatin and a decrease in myostatin and Axin2. The expression of FAK was increased and of MuRF-1 and atrogin-1 decreased only in the control group, whereas Ankrd2 expression was enhanced only in diabetic rats. In the soleus muscle caused similar changes in both groups: increase in FAK and MGF and decrease in Wnt7a, MuRF-1, atrogin-1, and myostatin. Differences between groups were observed only in the increased expression of follistatin in diabetic animals and decreased Ankrd2 expression in the control group. So, insulin deficiency does not impair the overload-induced hypertrophic response in soleus and EDL muscles. However, different mechanisms seem to be involved in the comparable hypertrophic responses of skeletal muscle in control and diabetic animals.

## Introduction

Type 1 diabetes (T1DM) is associated with marked changes in skeletal muscle morphology, electrical, and contractile properties and metabolism leading to impaired muscle function (Cotter et al. 1989, 1993; Cameron et al. 1990; McGuire et al. 2001). Recovery from injury and skeletal muscle regeneration are impaired in diabetes (Vignaud et al. 2007; Krause et al. 2011, 2013). Under these conditions, skeletal muscle displays a shift in the fiber types toward more oxidative or slow-twitch phenotypes (Armstrong et al. 1975). Growth and development of skeletal muscle are also impaired in T1DM (Krause et al. 2009; D’Souza et al. 2013), resulting in reduced myofiber diameter (Andersen et al. 1997, 2004) along with lowered capillary density and deficient angiogenesis (Leinonen et al. 1982; Krause et al. 2009; Rennert et al. 2014). These alterations together are diagnosed as diabetic myopathy (Krause et al. 2009; D’Souza et al. 2013).

The decrease in protein synthesis and increase in protein degradation lead to skeletal muscle mass loss and atrophy, a common feature of T1DM (Barazzoni et al. 2004; Krause et al. 2009). PI3K-Akt-mTOR is the major signaling pathway involved in maintaining skeletal muscle trophism and growth in response to hormones (e.g., testosterone, insulin and IGF-1) (Sukho 2003; Bolster et al. 2004; Basualto-Alarcón et al. 2013; Egner et al. 2013; Schiaffino et al. 2013) and nervous stimuli (Kern et al. 2014). Activation of this pathway increases protein synthesis and induces muscle hypertrophy (Taha and Klip 1999; Bodine et al. 2001; Miyazaki and Esser 2009; Yu and Baylies 2013) but its activity is attenuated in streptozotocin-induced diabetic animals due to reduced plasma levels of insulin and IGF-1 (Like and Rossini 1976; Yang et al. 1990). These animals also display a short-term increase in the proteolytic activity in the soleus and extensor digitorum longus (EDL) muscles 1–3 days after entering into the diabetic state returning to control levels after 5 days (Pepato et al. 1996). Hyperglycemia has been associated to reduced skeletal muscle mass (Oku et al. 2001; Russell et al. 2009) and in long-term leads to increased production of reactive oxygen species (ROS) and of advanced glycation end products (AGEs) that exacerbate the loss of skeletal muscle mass (Baynes 1991; Riboulet-chavey et al. 2006; Grzelkowska-Kowalczyk et al. 2013).

Several diabetes complications are caused by atrophic myopathy (D’Souza et al. 2013) that leads to reduced glucose homeostasis (DeFronzo et al. 1981a,b). Muscle weakness and limited mobility in long-term diabetes have been associated with incapacitation for routine physical activities such as walking, climbing stairs, and running (Andersen 2012). Decreasing mobility is also associated with high incidence of other morbidities, disabilities and mortality (Pahor et al. 2014). Therefore, improvements in skeletal muscle health and trophism are beneficial for glucose homeostasis (D’Souza et al. 2013) and preservation of the capacity of diabetic patients for physical activities and so to reach a better clinical condition.

Experimental models of muscle overload have been widely used to study skeletal muscle hypertrophy (Goldberg 1968; Armstrong et al. 1979). These models provide a large hypertrophic response in a short period of time, allowing the researcher to investigate hypertrophy in a way that is not possible in a low/moderate muscle hypertrophic condition (Lowe and Alway 2002).

The information above led us to examine the effects of overload-induced hypertrophy in EDL (fast-twitch) and soleus (slow-twitch) muscles from streptozotocin-induced diabetic rats. The rats were rendered diabetic for 30 days and then submitted to muscle overload protocol for further 7 or 30 days. Hypertrophy of EDL and soleus muscles was evaluated by measuring fiber cross-sectional areas (CSA) and dry and wet muscle weight. Strength and contractile properties such as maximum isotonic contractions (muscle twitch) and tetanic forces were also measured. Contents of total and phosphorylated Akt and rpS6 proteins, after 7 and 30 days of overload, and mRNA levels, after 7 days, of several genes associated to signaling pathways regarding skeletal muscle hypertrophy were determined.

## Methods

### Animals

Male Wistar rats (200 ± 50 g, at least six animals per group) were obtained from the Institute of Biomedical Sciences, University of São Paulo, and maintained in groups of three with water and food ad libitum in a room with 12/12 h light–dark cycle at 22°C. All experimental procedures were approved by the Animal Ethics Committee of the Institute of Biomedical Sciences of the University of São Paulo (CEUA – USP) and were carried out in agreement with the Guide for the Care and Use of Laboratory Animals (Institute of Laboratory Animal Resources, National Academy of Sciences, Washington, DC) and the principles of the Brazilian College of Animal Experimentation (COBEA).

### Induction of diabetes mellitus

Diabetes *mellitus* was induced by injection of 65 mg/kg b.w. streptozotocin dissolved in citrate buffer (pH 4.5) into the caudal vein (Ungvari et al. 1999). Control animals received the same volume of citrate buffer. Blood glucose levels above 400 mg/dL (22.2 mmol/L) after 24-h of the streptozotocin injection confirmed the diabetic state. Glucose levels were weekly measured to ensure that the animals remained diabetic up to the experiment being performed. Similar procedure was used in our previous studies (Vitzel et al. 2013). Animals in diabetic state for 30 days were submitted to surgery for skeletal muscle overload induction.

### Overload protocol of EDL and soleus muscles

To induce soleus muscle hypertrophy, unilateral synergistic tenotomy of the gastrocnemius muscles was performed as previously described (Goldberg 1972; Armstrong et al. 1979; Owino et al. 2001). Before surgery, animals were anesthetized by a single intraperitoneal injection of xylazine (10 mg/kg b.w.) and ketamine (90 mg/kg b.w.). An incision was made in the lower limb, exposing muscles, and tendons. The Achilles tendon was carefully separated in portions of gastrocnemius and soleus muscles, and then severed ensuring that no damage to the vasculature, nerves, and surrounding muscles occurred, leaving the soleus muscle insertion intact. All procedures were performed under aseptic conditions. The contralateral limb was used for sham surgery, in which soleus and gastrocnemius tendons were identified but not severed.

To induce EDL muscle overload, in a different set of animals, unilateral synergistic ablation of the tibialis anterior muscle was carried out as previously described (Rosenblatt and Parry 1992; Hamilton et al. 2010). The contralateral limb was submitted to sham surgery. Immediately after surgery, animals were intraperitoneally injected with gentamicin (40 mg/kg b.w.).

Animals remained under muscle overloading during 7 or 30 days. The unilateral ablation and tenotomy are advantageous to allow paired comparison between sham and overloaded muscles avoiding inaccuracies of the use of different animals (Thomson and Gordon 2006).

### Analysis of skeletal muscle contractile function

Evaluations of skeletal muscle contractile function were performed as previously described (Bassit et al. 2010; Pinheiro et al. 2010, 2011, 2012a). The animals were anesthetized by intraperitoneal injection of sodium pentobarbital (75 mg/kg b.w.) and both hindlimbs were fixed on an acrylic platform. The stimulated limb was placed on the platform with the hip joint at 120°–140° angle and the knee joint at a 120°–140° angle. A hook was placed under the Achilles tendon for soleus muscle measurements, or under the distal tendon of EDL muscle and connected to a force transducer (Grass Technologies, West Warwick, RI). Skin was removed and the sciatic nerve accessed. A platinum electrode was placed at the nerve. The fascia was separated from the muscles and all the synergistic and antagonist muscles were tenotomized to avoid any interference in force measurements. Muscle contractions were induced by electrical stimulation using symmetrical monophasic square waves with the MultiStim System D330 (Digitimer Ltd, Welwyn Garden City, Hertfordshire, UK). The resting length (L0) of the target muscle was adjusted to obtain maximum tension (the ankle joint approximately at a 90° angle) upon stimulation by regulating the traction of the hook coupled to the tendon and force transducer. Electrical stimulation-induced muscle contraction pulls the hook placed in the muscle tendon. For determination of the muscle twitch force, the stimulus consisted of 500 *μ*s pulse duration at 1 Hz with adjusted voltage to produce maximum force. To determine the tetanic force, electrical stimulus frequency was increased to 100 Hz. Muscle twitch force (maximum tension generated during twitch) and tetanic force (maximum tension generated) were recorded using the AqDados® software (version 4.16, Lynx Tecnologia Eletrônica Ltda, São Paulo, Brazil). The muscle strength and contractile properties were analyzed using the AqAnalysis® software (version 4.16, Lynx Tecnologia Eletrônica Ltda, São Paulo, Brazil). The same measurements were carried out in our previous studies (Pinheiro et al. 2011, 2012b).

### Histological analysis

Evaluation of fiber CSA was performed as previously described (Bodine and Baar 2012). Ten *μ*m thick sections from EDL and soleus muscles of the midportion region were obtained using a cryostat (Leica CM3050 S; Leica Microsystems, Nussloch GmbH, Germany) and stained with hematoxylin and eosin (HE) for fiber CSA examination. Sections were imaged using an upright microscope with camera (Nikon DXM 1200; Nikon Instruments, Melville, NY). Digitized images were analyzed using the Image-Pro Plus Software (Media Cybernetics, Silver Spring, MD) in a blinded manner. Similar analysis was performed in our previous studies (Pinheiro et al. 2011).

### Western blotting

Proteins were extracted in buffer containing Tris–HCl (100 mmol/L), pH 7.5; EDTA (10 mmol/L); sodium pyrophosphate (10 mmol/L); sodium fluoride (0.1 mmol/L); sodium orthovanadate (10 mmol/L); phenylmethylsulfonyl fluoride (PMSF) (2 mmol/L); and aprotinin (10 *μ*g/mL). The muscle extracts were sonicated for 30 s at 4°C. The homogenates were centrifuged at 12,000 g for 20 min at 4°C. The supernatants were treated with Triton X-100 (1%) and immediately transferred to a −80°C freezer for storage until western blotting analysis being performed. Bradford method was used to determine the total protein content in an aliquot from the supernatant. Laemmli buffer was added to the samples, followed by a 95°C heating for 5 min and sodium dodecyl sulfate polyacrylamide gel electrophoresis (SDS-PAGE). After protein transfer to a nitrocellulose blotting membrane and nonspecific sites blocking with free-fat milk, the membranes were incubated overnight at 4°C with primary antibodies against total Akt and ribosomal protein S6 (rpS6), phospho-Akt (Thr^308^) and phospho-rpS6 (Ser^244/240^) (Cell Signaling Technology, Beverly, MA) followed by treatment with a secondary HRP-conjugated antibody (Millipore, Temecula, CA) for 60 min at room temperature. Similar analysis was performed in our previous studies (Vitzel et al. 2013).

### Real-time polymerase chain reaction (RT-PCR)

Total RNA was extracted from skeletal muscles using RNeasy RNA isolation kit (Qiagen Inc, Valencia, CA) according to the manufacturer’s protocol. The expression of the genes was determined by RT-PCR. Total RNA quality and quantity were determined using a Nanodrop spectrophotometer (Thermo Scientific Nanodrop; Nanodrop Technologies, Wilmington, DE) and it was reverse transcribed with oligo(dT) using cDNA Synthesis Kit containing RevertAid M-MuLV reverse transcriptase (Invitrogen/Life Technologies, Carlsbad, CA). The cDNA levels of the genes were quantified using the SYBR green qPCR kit. RT-PCR was performed in a Rotor-Gene 3000 (Corbett Robotics, Australia). The mRNA content of the genes was normalized to Hypoxanthine Phosphoribosyltransferase 1 (HPRT1) mRNA levels. The following genes were evaluated: FAK – Focal Adhesion Kinase; Wnt 7a – Wingless-Type MMTV Integration Site Family, Member 7A; MuRF-1 – Muscle RING-Finger protein-1; IGF-1 Eb (MGF) – Insulin-like growth factor 1 Eb-peptide (mechano growth factor); Ankrd2 – Ankyrin repeat domain 2. The primers sequences used in the experiments are displayed in the Table1.

**Table 1:** Primers sequences of the genes which expressions were measured by RT-PCR

	Primer sequence (forward)	Primers sequence (reverse)
FAK	5′-AAGGAGCACCTCTCAAACCG-3′	5′-CATCGCTCCGACAGCATTTG-3′
Wnt 7a	5′-GCGCTCTAGGACAGTCTCCA-3′	5′-GGGGCAATCCACATAGCCTG-3′
MuRF-1	5′-GGACCGGCATGGGGTGTACG-3′	5′-TTTCTGCAGGGGCCGACTGG-3′
Atrogin-1	5′-CGGCACCTTCGTGAGCGACC-3′	5′-GTGCAGATATCCATGGCGCTCCT-3′
IGF-1 Eb (MGF)	5′-GCTTGCTCACCTTTACCAGC-3′	5′-AAGTGTACTTCCTTTCCTTCTC-3′
Myostatin	5′-TACCACGGAAACAATCATTACCAT-3′	5′-TGCCATCCGCTTGCATT-3′
Follistatin	5′-AGCGAGTGTGCCATGAAG-3′	5′-GAGTGGAAGAGATAGGGAAGC-3′
Ankrd2	5′-TGATGCCGTGAGACTCAACC-3′	5′-TTAGCCATCATGTCTGCCCC-3′
Axin 2	5′-CTCAGCAAAAAGGGAAATTACAGGTAT-3′	5′-ACTGTCTCGTCGTCCCAGATCTC-3′
HPRT1	5′-GCGAAAGTGGAAAAGCCAAGT-3′	5′-GCCACATCAACAGGACTCTTGTAG-3′

FAK – Focal Adhesion Kinase; Wnt 7a, Wingless-Type MMTV Integration Site Family, Member 7A; MuRF-1, Muscle RING-Finger protein-1; IGF-1Eb (MGF), Insulin-like growth factor 1Eb-peptide (mechano growth factor); Ankrd2, Ankyrin repeat domain 2; HPRT1, Hypoxanthine Phosphoribosyltransferase 1.

### Statistical analysis

The results are presented as mean ± SEM. Student’s *t* test was used for the comparisons of the responses in muscle dry weight and twitch and tetanic forces between the diabetic and the control groups. Two-way analysis of variance (ANOVA) followed by Bonferroni post test was used for all analysis. The Bonferroni post test was used for comparisons between contralateral versus hypertrophied muscles of the same group, and contralateral versus contralateral muscles and hypertrophied versus hypertrophied muscles of different groups. As indicated in the text and the Figure legends, two-way ANOVA only was also used for comparison between diabetic versus control rats considering both contralateral and hypertrophied muscles and for comparison between diabetic and control hypertrophied muscles versus diabetic and control contralateral muscles. Grubb’s test was used to exclude outliers. Differences between values were considered statistically significant for *P* < 0.05. All results were analyzed using the GraphPad Prism 5.0 statistical software (GraphPad Software, San Diego, CA). Analysis of fibers CSA was performed as previously described using the 95% confidence interval of the mean (Pinheiro et al. 2011).

## Results

### Muscle weight

After 30 days of overload, the increase in EDL muscle wet weight per tibia length was of 10.6 mg/cm (39%) in diabetic animals and of 24.7 mg/cm (52%) in controls (Table2). Soleus muscle presented an increase in absolute wet weight per tibia length of 18 mg/cm (59%) in diabetic animals and 21.6 mg/cm (55%) in controls. The wet weight of the contralateral muscles (on the tibia length basis) of the control group was higher than that of the muscles from diabetic animals. Although the absolute wet weight of the muscles is different, being the muscles from diabetic rats smaller, the hypertrophic response in percentage was comparable. In fact, hypertrophic response (as indicated by percentage increase in dry weight) of the EDL and soleus muscles was not different between diabetic and control rats.

**Table 2:** Hypertrophic effects of overload on the EDL and soleus muscles

Measurements	Diabetic group		Control group	
	Hypertrophied	Contralateral	Hypertrophied	Contralateral
EDL Muscle				
Wet weight (mg)	146.2 ± 7.2*,###	110.4 ± 7§§§	297 ± 15.7***	194 ± 9.2
Dry weight (mg)	38.7 ± 1.9***,###	30.6 ± 1.9§§§	74.2 ± 2.7***	53.2 ± 2
Wet weight per tibia length (mg/cm)	37.5 ± 1.8**,###	26.9 ± 1.6§§§	71.6 ± 3.6***	46.9 ± 2.3
Dry weight per tibia length (mg/cm)	9.6 ± 0.4**,###	7.5 ± 0.4§§§	17.9 ± 0.6***	12.9 ± 0.5
Soleus Muscle				
Wet weight (mg)	182.9 ± 7.7***,###	114.4 ± 4.8§§§	244.5 ± 11.7***	155.6 ± 4.9
Dry weight (mg)	37.2 ± 2.5*,###	26.1 ± 1.2§§§	61.7 ± 3.9***	45.2 ± 2.5
Wet weight per tibia length (mg/cm)	48.2 ± 1.9***,###	30.2 ± 1.3§§	60.5 ± 2.8***	38.9 ± 1.2
Dry weight per tibia length (mg/cm)	9.9 ± 0.5*,###	6.5 ± 0.4§§§	15.1 ± 0.9***	11.1 ± 0.6

Results are expressed as mean ± SEM of at least six animals.

* *P* < 0.05

** *P* < 0.01

*** *P* < 0.001 hypertrophied versus contralateral

### *P* < 0.001 hypertrophied versus hypertrophied of different groups

§§ *P* < 0.01

§§§ *P* < 0.001 contralateral versus contralateral of different groups. Data were analyzed using two-way ANOVA followed by Bonferroni post test. EDL, extensor digitorum longus.

### Muscle forces

Absolute tetanic force was increased by 35% in the overloaded EDL muscle of the diabetic and the control groups. Specific tetanic force expressed on a muscle dry weight basis was elevated by 33% in diabetic and by 48% in control animals. Absolute twitch force was raised by 72% in diabetic and by 45% in control rats, whereas specific twitch force was raised by 17% in the diabetic and by 28% in the control groups. In the overloaded soleus muscle, absolute tetanic force was increased by 107% in the diabetic and by 98% in the control groups. Specific tetanic force was increased by 107% in diabetic and by 40% in control animals. The diabetic group presented an increase of 94% in the specific twitch force, whereas in controls it was of 65%. Absolute twitch force was increased by 94% in the control group but no significant difference was found in the overloaded soleus muscle from the diabetic group (Table3).

**Table 3:** Absolute and specific forces of the soleus and EDL muscles

Measurements	Diabetic group		Control group	
	Hypertrophied	Contralateral	Hypertrophied	Contralateral
EDL muscle				
Absolute tetanic force (mN)	3147.5 ± 377.6*,##	1985.9 ± 283.7§§	4972.2 ± 437.4	3686.2 ± 270.3
Specific tetanic force (mN/mg)	88.1 ± 7.1*	65.9 ± 6.9	77.8 ± 6.5*	52.5 ± 4.4
Absolute twitch force (mN)	1217.9 ± 74.9**,##	705 ± 102.9§	1572.5 ± 143.6*	1085.1 ± 58.7
Specific twitch force (mN/mg)	34.5 ± 1.9###	29.4 ± 2.0§§	22.1 ± 1.7	17.3 ± 0.9
Soleus muscle				
Absolute tetanic force (mN)	1415.3 ± 95.2#	681.5 ± 156.7	2451.9 ± 488.7**	1236.2 ± 89.7
Specific tetanic force (mN/mg)	42.3 ± 2.6***,#	20.3 ± 3.9	32.5 ± 2.5*	23.1 ± 1.0
Absolute twitch force (mN)	459.5 ± 63.8#	342.7 ± 17.5	851.9 ± 83.7***	438.1 ± 70.8
Specific twitch force (mN/mg)	22.9 ± 2.7***,#	11.8 ± 0.7	14.2 ± 2.0*	8.6 ± 0.9

Tetanic force was determined at 100 Hz of electrical stimulus frequency. Muscle twitch (isotonic contraction) was determined at 1 Hz of electrical stimulus frequency. Data are expressed as mean ± SEM of at least six animals.

* *P* < 0.05

** *P* < 0.01

*** *P* < 0.001 hypertrophied versus contralateral

# *P* < 0.05

## *P* < 0.01

### *P* < 0.001 hypertrophied versus hypertrophied of different groups

§ *P* < 0.05

§§ *P* < 0.01 contralateral versus contralateral of different groups. Data were analyzed using two-way ANOVA followed by Bonferroni post test. EDL, extensor digitorum longus.

### Fiber CSA

**Figure 1:**
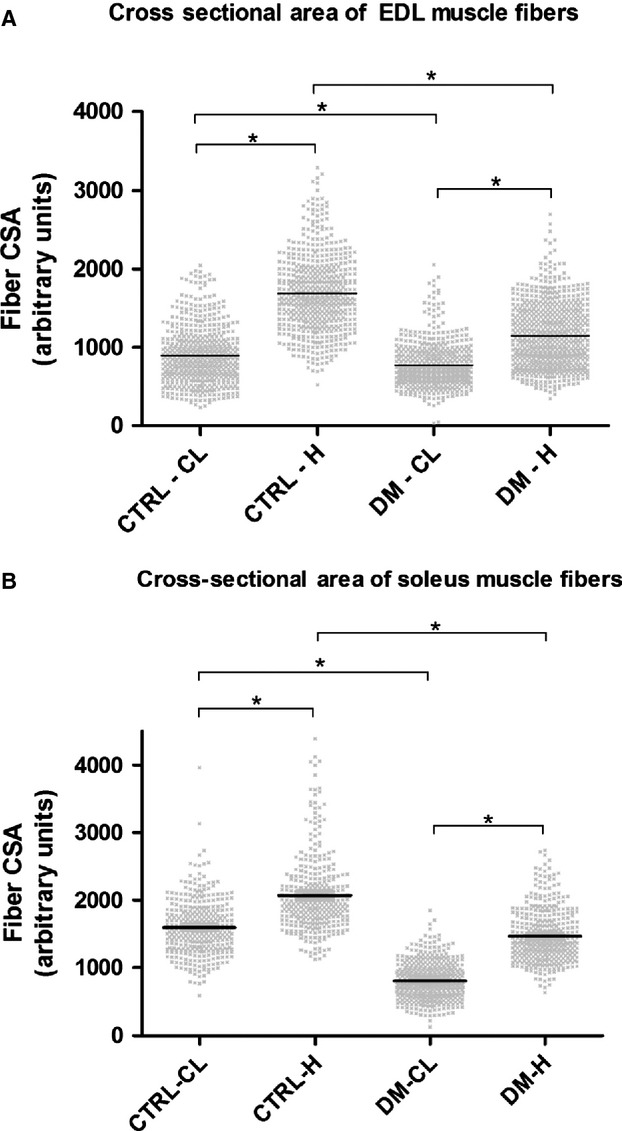
Cross-sectional areas of EDL and soleus muscles after 30 days of overload. Panel (A) Cross-sectional areas of the EDL muscle fibers. Panel (B) Cross-sectional areas of the soleus muscle fibers. Results are expressed as mean ± SEM of six animals (120 fibers per muscle). CTRL-CL = Control group, contralateral muscle, CTRL-H = Control group, hypertrophied muscle, DM-CL = Diabetic group, contralateral muscle, DM-H = Diabetic group, hypertrophied muscle. *P < 0.05 using the 95% confidence interval of the mean.

After 30 days of overload, the average fiber CSA of the EDL muscle showed an increase of 48% in the diabetic and of 88% in the control groups. In the soleus muscle, the increase in CSA was of 83% and 30% in diabetic and control rats, respectively (Fig.1).

### Western blotting

**Figure 2:**
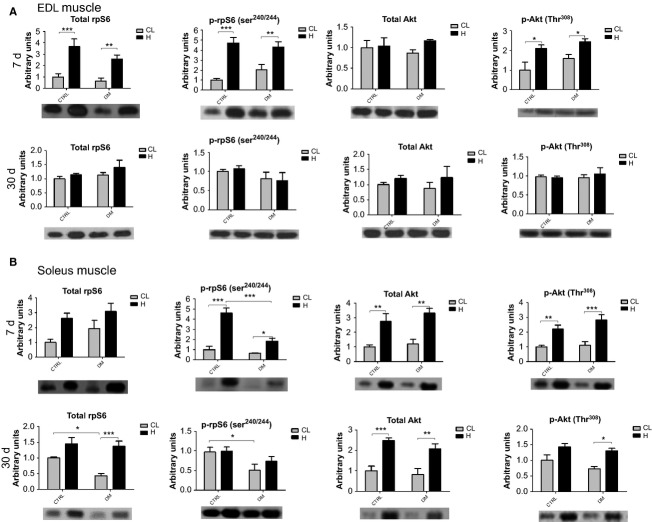
Western Blots of EDL and soleus muscles after 7 and 30 days of overload. Panel (A) Quantitative analysis of western blotting and representative images of total rpS6 and Akt and phosphorylated rpS6^240/244^ and Akt^308^ of the EDL muscle after 7 and 30 days of overload. Panel (B) Quantitative analysis of western blotting and representative images of total rpS6 and Akt and phosphorylated rpS6^240/244^ and Akt^308^ of the soleus muscle after 7 and 30 days of overload. Data are expressed as mean ± SEM of at least six animals. *P < 0.05, **P < 0.01, ***P < 0.001. Also using two-way ANOVA only, total rpS6 protein was different between diabetic and control hypertrophied muscles versus diabetic and control contralateral muscles (*P* < 0.05). CTRL, Control group; DM, Diabetic group; CL, Contralateral muscle; H, Hypertrophied muscle.

In the EDL muscle (*n* = 6), after 7 days of overload, total Akt content was not altered, however, phospho-Akt content was increased by 2.1-fold in the control and by 1.5-fold in the diabetic groups. An increase in the total content of rpS6 was observed in the control and in the diabetic groups (by 3.6- and fourfold, respectively). The phospho-rpS6 content was increased by 4.8-fold in the control and by 2.1-fold in the diabetic groups (Fig.2). After 30 days of overload, total rpS6 and Akt and phosphorylated rpS6 and Akt returned to basal levels in the EDL muscle (Fig.2).

In the soleus muscle (*n* = 6), after 7 days of overload, total Akt content was increased by 2.7-fold in both groups. Phospho-Akt content was increased by 2.2-fold in control and by 2.5-fold in diabetic animals. Total content of rpS6 was not different between the contralateral muscles of the control and the diabetic groups. The overload caused a similar increase in the levels of this protein in both groups. An increase in the content of phospho-rpS6 was observed in the diabetic and the control groups (by 2.9- and 4.7-fold, respectively) (Fig.2). After 30 days of overload, total Akt was increased by 2.5-fold in both groups, whereas phospho-Akt was increased by 1.7-fold in the diabetic group only (Fig.2). The muscle overload raised total rpS6 content by 3.1-fold in diabetic rats but it was not significantly changed in the control group. Phospho-rpS6 content was not altered by the overload. However, there was a reduction of 47% in the content of this protein in the contralateral muscle of the diabetic animals when compared to controls.

### mRNA expression of FAK, Wnt7a, MuRF-1, atrogin-1, IGF-1 Eb (MGF), myostatin, follistatin, Ankrd2, and Axin2

**Figure 3:**
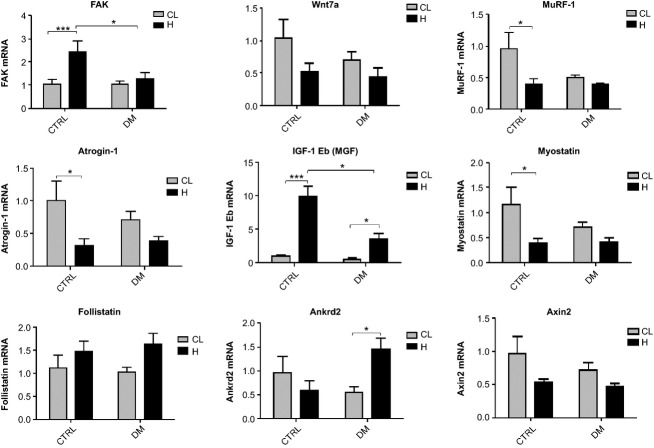
mRNA expression of several genes as measured by RT-PCR in the EDL muscle. Results are expressed in arbitrary units and presented as mean ± SEM of 6 animals. Data were analyzed using two-way ANOVA followed by Bonferroni post test. *P < 0.05, ***P < 0.001. As indicated by the two-way ANOVA only (*P* < 0.05), the expressions of the following genes were different between the diabetic versus the control groups considering both the contralateral and hypertrophied muscles: MuRF-1 and atrogin-1. Also using two-way ANOVA only (*P* < 0.05), the expressions of the following genes were different between diabetic and control hypertrophied muscles versus diabetic and control contralateral muscles: myostatin, follistatin and Axin2. EDL, extensor digitorum longus; CL, Contralateral muscle; H, Hypertrophied muscle; DM, Diabetic group; CTRL, Control group.

mRNA content of the genes was evaluated after 7 days of functional overload in the EDL and soleus muscles. The hypertrophied EDL muscle of diabetic rats presented increased expression of FAK (24%) when compared to the contralateral muscle whereas in the control group the expression of this gene was 133% higher. The mRNA content of MGF was enhanced by sixfold in the diabetic and by 10-fold in the control groups in EDL muscle due to overload stimulus. mRNA content of MuRF-1 was reduced by 59% in the control group as observed by Bonferroni post test. Using two-way ANOVA only, the diabetic state also reduced MuRF-1 expression. Atrogin-1 mRNA expression was reduced by 68% in the control but it was not changed in the diabetic groups. However, as indicated by two-way ANOVA only, diabetes reduced atrogin-1 expression in EDL muscle. Expression of myostatin was reduced by 67% in the control group but no significance was founded by the post test in the diabetic group. Analysis by two-way ANOVA showed a significant reduction in the expression of most genes by hypertrophy. Bonferroni post test did not show any difference in mRNA expression of follistatin between groups. However, muscle hypertrophy presented a significant effect on increasing follistatin mRNA levels as pointed out by two-way ANOVA. Ankrd2 mRNA content was increased by 165% in diabetic but showed no significant change in control rats. Hypertrophic stimulus decreased mRNA expression of Axin2 in the overloaded EDL muscle of both groups as indicated by two-way ANOVA only (Fig.3).

**Figure 4:**
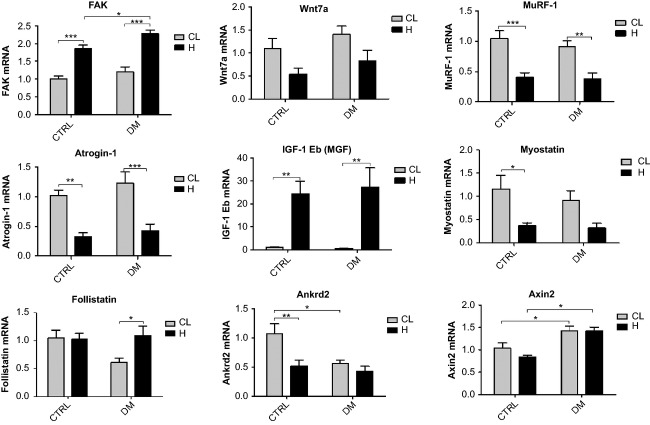
mRNA expression of several genes as measured by RT-PCR in the soleus muscle. Results are expressed as arbitrary units and presented as mean ± SEM of six animals. Data were analyzed using two-way ANOVA followed by Bonferroni post test. *P < 0.05, **P < 0.01, ***P < 0.001. As indicated by two-way ANOVA only (*P* < 0.05), the expression of the following genes was different between diabetic versus control groups considering both contralateral and hypertrophied muscles: Axin2. Also using two-way ANOVA only (*P *< 0.05), the expressions of the following genes were different between diabetic and control hypertrophied muscles versus diabetic and control contralateral muscles: Wnt7a and myostatin. CL, Contralateral muscle; H, Hypertrophied muscle; DM, Diabetic group; CTRL, Control group.

Hypertrophied soleus muscle presented higher mRNA levels of FAK as compared to the contralateral muscle of the diabetic (88%) and the control (83%) groups. Wnt7a expression was significantly reduced by hypertrophy, as indicated by two-way ANOVA only. The increase in MGF mRNA content induced by overload stimulus in soleus muscle was remarkable even compared with that observed in the EDL: by 52- and 24-fold in diabetic and control rats, respectively. There was a reduction in the expression of MuRF-1 and atrogin-1 in the hypertrophied muscle: MuRF-1 by 58% in the diabetic group and by 62% in controls; atrogin-1 by 66% in the diabetic group and by 68% in controls. Myostatin expression was significantly lower (68%) in the hypertrophied soleus muscle of the control group. Two-way ANOVA indicated a decreasing effect of hypertrophy on mRNA content of myostatin in soleus muscle. Follistatin mRNA levels presented a 79% increase due to the overload in soleus muscle from diabetic animals only. Ankrd2 mRNA content was decreased by 51% in controls due to hypertrophy and by 47% in the contralateral muscle of diabetic animals when compared to contralateral soleus muscle of the control group. Increased mRNA expression of Axin2 due to the diabetic state was also found by two-way ANOVA only (Fig.4).

## Discussion

Advanced glycation end products are generated under hyperglycemic conditions and can impair several signaling pathways such as protein kinase C (PKC), nuclear factor kB (NF-kB) and transforming growth factor beta (TGF-*β*) (Brownlee 2001; Riboulet-chavey et al. 2006), leading to a decrease in the hypertrophic signal. Impaired hypertrophy of the skeletal muscle has been associated with low-mitochondrial biogenesis and an increase in AMPK activity that attenuate S6K1, 4E-BP1, and eEF2 activities (Thomson and Gordon 2005; Thomson et al. 2008). Impaired regeneration of the skeletal muscle and low-force production are also observed in diabetic mice (Vignaud et al. 2007). Hypertrophy is lowered in insulin resistance state due to less activation of mTOR as observed in Zucker rats (Paturi et al. 2010). Mice fed a hyperlipidic diet show less activation of Akt and p70S6K1 after functional overload in the plantaris muscle (Sitnick et al. 2009). It is also important to mention that diabetic condition is associated with low angiogenesis and capillary density in mice skeletal muscle (Emanueli et al. 2002; Kivelä et al. 2006, 2008). However, despite the mentioned vascular impairment, we observed similar percentage increase in skeletal muscle hypertrophic response in both diabetic and control rats.

Specific force was more elevated in EDL and soleus muscle of diabetic rats. Sarcoplasmic concentration of calcium has been reported to be increased in diabetic animals (Nakagawa et al. 1989; Eshima et al. 2013). Sarco/endoplasmic reticulum Ca^2+^-ATPase (SERCA) inhibition occurs by high contents of reactive oxygen species (Andrade et al. 1998, 2001), which are significantly increased in the skeletal muscles from diabetic animals (Gumieniczek et al. 2001; Aragno et al. 2004; Bravard et al. 2011). Excess of calcium in the sarcoplasm is likely to overwhelm calcium pumps reuptake activity and accumulation of this ion in the sarcoplasmic reticulum then occurs (Eshima et al. 2013). Under this condition, the intrinsic response of the skeletal muscle contraction may become more intense (Nakagawa et al. 1989).

Phospho-Akt and rpS6 contents were increased in the EDL muscle after 7 days of overload. These results corroborate previous studies (Thomson and Gordon 2006; Miyazaki et al. 2011) performed in plantaris muscles of rats also after 7 days of a similar muscle overload protocol used herein. When the EDL muscle was submitted to 30 days of overload, Akt and rpS6 did not present any significant difference in the total or phosphorylated proteins levels, indicating that this protein synthesis pathway activity returned to pre-overload values. Peterson and coworkers (Peterson et al. 2008) showed that the overload-induced response on the plantaris muscle also returns to pre-overload values after 21 days.

After 7 days of overload in the soleus muscle, the contents of total and phospho-Akt and rpS6 were increased in both groups. After 30 days of overload in soleus muscle, the contents of total Akt in controls and of total rpS6 and phospho-Akt in the diabetic group remained increased. Thus, protein synthesis pathway may have sustained an elevated activity under these conditions, even after 30 days of overload, particularly in the soleus muscle of diabetic animals. Gastrocnemius muscle presents, after 20 days of overload, elevated contents of phosphorylated mTOR, p70S6K1, and Akt confirming a persistent activation of this signaling pathway during a prolonged period of time (Ochi et al. 2010). The persistent activation of the protein synthesis pathway that lasted until to 30 days probably occurred due to higher susceptibility of the soleus muscle to a mechanical load-induced injury in diabetic rats (Copray et al. 2000). In spite of the observed difference between soleus and EDL in the persistence of protein synthesis pathway activation, both muscles from control and diabetic animals presented corresponding hypertrophic responses after 30 days of overload.

The occurrence of hypertrophy in soleus, plantaris and gastrocnemius muscles in response to 8 weeks of resistance exercise has previously been shown in diabetic rats (Farrell et al. 1999). The authors postulated that hypertrophy occurred because the IGF-1 increase was able to compensate for the low-plasma insulin levels. In fact, IGF-1 has been shown to play a central role in the stimulation of protein synthesis pathway induced by overload (Coleman et al. 1995; Bodine et al. 2001; Rommel et al. 2001; Schiaffino and Mammucari 2011). IGF-1 administration stimulates cell proliferation and enhances myogenic differentiation and hypertrophy in cultured muscle cells (Engert et al. 1996) and transgenic mice (Coleman et al. 1995). These effects of IGF-1 could produce a satisfactory hypertrophic response in skeletal muscle from diabetic rats. We observed a large increase in IGF-1 Eb (MGF) expression by overload both in control and diabetic animals, but it may not account alone for their similar hypertrophic response. For instance, we showed herein that MGF gene expression is almost twice as high in the control as compared to the diabetic groups in the EDL muscle, however, hypertrophic response was similar between groups. In fact, muscle hypertrophy due to overload does not depend exclusively on IGF-1 stimulus (Spangenburg et al. 2008; Klossner et al. 2009) and it can also occur without Akt activation (Miyazaki et al. 2011). Therefore, MGF may not be the major player in the similar hypertrophic responses observed in the diabetic and control groups. For instance, the MGF that is locally produced in skeletal muscle submitted to mechanical stress causes hypertrophy by activating the IGF receptors (Hill and Goldspink 2003) and, as discussed below, FAK stimulates p70S6K1 without Akt activation (Klossner et al. 2009). We showed herein that 7 days of overload caused noticeable variation in the mRNA contents of several genes associated to the control of muscle mass, and each experimental group presented a specific response.

Fluck and coworkers stated that FAK plays an important role in skeletal muscle hypertrophy (Flück et al. 1999). FAK has the ability to phosphorylate p70S6K1 in Tyr 397, independently of Akt and mTOR (Klossner et al. 2009). Hypertrophy mediated by IGF-1 requires the participation of FAK in C2C12 cells (Crossland et al. 2013). A decrease in the hypertrophic process after removal of FAK that affects mTOR-rpS6-eIF4F (eIF4F – eukaryotic initiation factor 4F) signaling pathway has been reported in C2C12 cells (Crossland et al. 2013). In the EDL muscle, FAK expression was increased by the overload in control but it did not change in diabetic animals. However, in soleus muscle, FAK was increased in both groups, being more pronounced in diabetic rats, suggesting a possible compensatory hypertrophic mechanism. This difference in FAK expression response to hypertrophy was also shown in other muscles such as the plantaris muscle (Gordon et al. 2001). Also, FAK activity is increased in young men after resistive training and was associated with muscle remodeling when its total content was diminished after bed rest (Li et al. 2013). Therefore, FAK signaling pathway may play a key role for the response of skeletal muscle hypertrophy in diabetic states.

Ankrd2 has been associated to muscle hypertrophy being triggered by mechanical signals (Tsukamoto et al. 2002; Kojic et al. 2004). Herein, we showed different responses of Ankrd2 expression in the overloaded EDL and soleus muscles: in the EDL, an increase was observed in the diabetic group only, whereas a decrease was found in the soleus muscle from the control group only.

FAK and Ankrd2 play an important role for muscle mechanotransduction. In the soleus muscle, when FAK expression was increased, Ankrd2 expression was unchanged or even lowered. Apparently, overload-induced hypertrophy of the soleus muscle relies more on FAK signaling rather than Ankrd2, regardless the diabetic condition. EDL muscle from control animals showed the same feature observed for soleus muscle. On the other hand, in the EDL muscle of diabetic animals, FAK expression was unchanged and Ankrd2 increased upon overload. Thus, mRNA expression in the EDL muscle suggests that under diabetic condition, FAK hypertrophic signaling was suppressed, being replaced by Ankrd2. A compensatory mechanism of FAK and Ankrd2 may ensure the muscle hypertrophic responses even in catabolic conditions such as diabetes.

Wnt7a binds to Frizzled7 (Fzd7) receptor and activates the Fzd7-PI3K-Akt-mTOR pathway, leading to skeletal muscle hypertrophy (Von Maltzahn et al. 2012a,b). Wnt signaling pathway also promotes hypertrophy through the canonical pathway, involving *β*-catenin and Axin2, that are activated during overload of the skeletal muscle (Armstrong and Esser 2005). We showed no change in gene expression of Wnt7a and Axin2 in the EDL muscle. However, in the overloaded soleus muscle of both groups, there was a decrease in Wnt7a expression that was not followed by changes in Axin2 mRNA content. The diabetic animals presented higher mRNA levels of Axin2 than controls, without any further alteration by the overload, indicating a possible modulation through *β*–catenin signaling pathway. Wnt7a nor Axin2 does not seem to be involved on overload-induced hypertrophy in this present study.

MuRF-1 and atrogin-1 are two muscle-specific E3 ubiquitin ligases that are increased in atrophic conditions and are involved in the ubiquitin binding and the consequent protein degradation by the 26S proteasome (Bodine and Baehr 2014). When the PI3K-Akt-mTOR pathway is activated, these proteins have their expression reduced due to inhibition by the upstream Forkhead box O3 (FOXO3) (Stitt et al. 2004). The decrease in the mRNA expression of MuRF-1 and atrogin-1 was observed after 7 days of overload in the soleus muscle of both groups. In contrast, the EDL muscle showed a reduction in mRNA levels of MuRF-1 and atrogin-1 only in the control group as indicated by two-way ANOVA. The diabetic group presented lower levels of MuRF-1 and atrogin-1 before muscle overload. Expression of MuRF-1 and atrogin-1 was increased after 1 day of overload, however, there was a reduction in the expression of the E3 ligases after 7 days corroborating our results (Baehr et al. 2014). Also, hypertrophy seems to occur when both synthesis and degradation of proteins are more elevated (Baehr et al. 2014). In control animals, reduction in the expression of MuRF-1 and atrogin-1, and a possible decrease in proteasome-dependent protein degradation, may be involved in the hypertrophic response to overload in soleus and EDL muscles. In diabetic animals, reduction in the expression of MuRF-1 and atrogin-1 was present in the soleus muscle, but it was lost in the EDL muscle, that had already a lower basal expression of these genes. Therefore, reduced expression of E3 ubiquitin ligases cannot be accounted as a mechanism associated to overload-induced hypertrophy in EDL muscle from diabetic rats.

Follistatin promotes muscle hypertrophy by inhibiting the repressive effects of myostatin on differentiation and growth of myogenic precursor cells and by increasing protein synthesis through mTOR-p70S6K1 pathway via Smad3-dependent mechanism (Winbanks et al. 2012). Myostatin is a negative regulator of skeletal muscle growth being associated with down-regulation of the Akt-mTOR signaling pathway and decreased phosphorylation of Akt, rpS6, p70S6K1, and 4E binding protein 1 (4E-BP1) (Amirouche et al. 2009; Rodriguez et al. 2014). Taking as a whole, follistatin increases muscle hypertrophic response and myostatin decreases it (Gilson et al. 2009). Myostatin expression was decreased due to overload in both muscles whereas follistatin expression was increased in the overloaded EDL and soleus muscles of the diabetic group only. These changes in follistatin and myostatin balance in diabetic animals may assist in the hypertrophic response herein observed.

Differences between EDL and soleus muscles regarding signaling pathways in the hypertrophic response remain to be elucidated. Ablation and tenotomy protocols lead remaining muscles to hypertrophy in a not fully comparable way. The load that tibialis anterior muscle presents on EDL muscle upon ablation is different from that which gastrocnemius muscle presents on soleus muscle upon tenotomy. Also, soleus muscle is an oxidative and postural muscle, and it is more recruited than the glycolytic EDL muscle. The variability between EDL and soleus muscles in response to overloading could also be associated to differences in blood perfusion, and this possibility still needs clarification.

**Figure 5:**
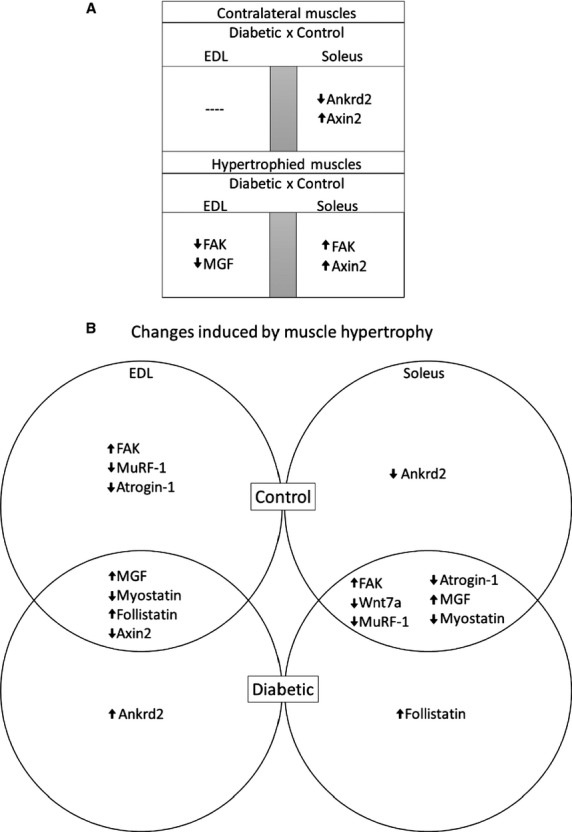
mRNA features of EDL and soleus muscles after 7 days of overload. Panel (A) Diagram of differences and similarities between mRNA expression of the genes of contralateral versus contralateral and hypertrophied versus hypertrophied EDL and soleus muscles from diabetic and control rats after 7 days of overload. Panel (B) Venn diagram of differences in mRNA expression of genes between contralateral versus hypertrophied EDL and soleus muscles from diabetic and control rats after 7 days of overload. FAK, Focal Adhesion Kinase; Wnt 7a, Wingless-Type MMTV Integration Site Family, Member 7A; MuRF-1, Muscle RING-Finger protein-1; MGF, Mechano growth factor; Ankrd2, Ankyrin repeat domain 2.

As comparable hypertrophic responses were found, different patterns of mRNA expression were involved leading to similar outcome. Regarding contralateral versus contralateral soleus muscles, there was a decrease in the Ankrd2 and an increase in Axin2 expression in the diabetic group, however, there was no alteration in the EDL muscle. When comparing hypertrophied versus hypertrophied muscles, EDL muscle showed a decreased expression of FAK and MGF in the diabetic group as compared to the control. In contrast, soleus muscle presented an increase in FAK and Axin2 expression in overloaded EDL muscle (Fig.5A).

The overloaded EDL muscle of control and diabetic animals showed an increase in MGF and follistatin expression and a decrease in myostatin and Axin2 mRNA levels. The expression of FAK was increased and of MuRF-1 and of atrogin-1 decreased only in the control group, whereas Ankrd2 expression was enhanced only in the diabetic group. The overloaded soleus muscle caused similar changes in control and diabetic rats: increased FAK and MGF and decreased in Wnt7a, MuRF-1, atrogin-1, and myostatin. Differences were observed only in the increased expression of follistatin in diabetic animals and the decreased Ankrd2 expression in the control group (Fig.5B). Both soleus and EDL muscles of control animals shared common features in expression of genes associated to hypertrophic mechanisms (e.g., increased FAK and IGF-1 Eb, and reduced Myostatin, MuRF-1, atrogin-1) upon overload. However, under diabetic state, this response was different for each muscle, nevertheless, achieving a similar hypertrophic response (in percentage increase) to control. Further experiments are necessary to fully elucidate, which specific pathways are involved in the hypertrophic response of skeletal muscle in diabetic state.

## Conclusion

Soleus and EDL muscles of diabetic rats submitted to functional overload for 30 days presented similar hypertrophic response when compared to controls. Muscle hypertrophy was accompanied by an increase in absolute tetanic and twitch forces. Regarding the protein synthesis pathway, phospho-Akt and phospho-rpS6 levels were elevated after 7 days of overload in both muscles, remaining increased for as long as 30 days in the soleus muscle only. Different expression patterns of several genes related to skeletal muscle hypertrophy were observed in both muscles of diabetic and control animals, indicating that different mechanisms seem to be involved. Our results indicate that insulin deficiency does not impair the overload-induced hypertrophic response of soleus and EDL muscles.
